# Pharmacist-led strategy to address the opioid crisis: The Medication Assessment Centre Interprofessional Opioid Pain Service (MAC iOPS)

**DOI:** 10.1177/17151635211045950

**Published:** 2021-09-21

**Authors:** Katelyn Halpape, Derek Jorgenson, Anan Ahmed, Kelly Kizlyk, Eric Landry, Radhika Marwah, Taylor Raiche, Amy Wiebe

**Affiliations:** College of Pharmacy and Nutrition (Halpape, Jorgenson, Ahmed, Kizlyk, Landry, Raiche, Wiebe) and the Department of Family Medicine (Marwah), University of Saskatchewan, Saskatoon, SK

## Background

One in 5 Canadians experience chronic pain, with increased prevalence in older adults, females, Indigenous peoples and populations affected by social inequities.^1^ Chronic pain is a complex biopsychosocial condition, and management is influenced by the intricate relationship between pain, mental health and physical well-being. Unmanaged chronic pain negatively affects all aspects of an individual’s life and society as a whole.^1^ Chronic pain management is challenging due to the number of people affected and the significant time and resources required to provide comprehensive, integrated care. Specialized care may be offered in interprofessional chronic pain clinics, but wait lists in many regions are exceptionally long, and in some areas, these clinics are nonexistent.^2^

The management of chronic pain has been complicated by the opioid crisis and the subsequent increased societal awareness of opioid use. Opioids play an important role in treating pain, but the risk of harm related to opioid use is also high. Between January 2016 and June 2020, there were more than 17,602 opioid-related deaths within Canada, and at least 1 pharmaceutical opioid was involved in 33% and 16% of opioid toxicity deaths among females and males, respectively.^3^ The 2017 Canadian Guideline for Opioids for Chronic Non-Cancer Pain provides recommendations regarding the safe and appropriate use of opioids to manage chronic pain.^4^ In addition, other strategies have been implemented to address the opioid crisis.^5^ Unfortunately, some of these strategies have created barriers for patients with chronic pain to access care, including difficulty obtaining opioid prescriptions.^1^ Health professionals must work collaboratively and in a patient-centred manner to develop individualized treatment plans, to provide education on how to minimize opioid risk and to offer support and follow-up.

In an effort to provide better access to interprofessional chronic pain management, the Medication Assessment Centre (MAC) has recently launched the Medication Assessment Centre Interprofessional Opioid Pain Service (MAC iOPS), using an innovative, pharmacist-led, interprofessional approach. This new service aims to use pharmacists’ unique knowledge and skills to implement and evaluate a new way to manage chronic pain in Saskatchewan. This article provides an overview of the MAC iOPS and offers insight into the establishment of this unique model of care.

## The MAC iOPS

The MAC, established in 2011, is a pharmacist-led patient care clinic, supported by the College of Pharmacy and Nutrition at the University of Saskatchewan (UofS). The MAC has collaborated with hundreds of family physicians and nurse practitioners (NPs) across Saskatchewan by providing comprehensive medication management for general medicine patients and cognitive behavioural therapy for insomnia.^6,7^ A pilot study found that 28% of individuals referred to the MAC for a general medication assessment were taking an opioid for chronic pain.^8^ These results highlighted an opportunity to focus on chronic pain management and opioid stewardship at the MAC, to better meet the needs of this population. In November 2019, with the support of funding from the Health Canada Substance Use and Addictions Program, patient care services at the MAC were expanded with the creation of the MAC iOPS, to offer interprofessional care to Saskatchewan residents living with chronic pain.

The MAC iOPS aims to

1. Optimize pain control, function and quality of life for individuals living with chronic pain;
2. Reduce the risk of medication-related harms for individuals taking prescribed medications for chronic pain; and
3. Improve primary care providers’ access to an interdisciplinary team with experience in managing chronic pain to support practice change in the areas of chronic pain and opioid prescribing.

## The MAC iOPS team

The MAC iOPS clinical team structure includes 2 full-time pharmacist positions, 1 part-time chronic pain physician and 1 part-time dietitian. The chronic pain physician has training as an anesthetist and currently practises in family medicine, with a long-standing practice in chronic pain management. Prior to working at the MAC iOPS, the pharmacists did not have formal postgraduate training in chronic pain management, but all were provided with professional development opportunities to prepare for this new role. For instance, each pharmacist spent time in an existing interprofessional chronic pain clinic, observing and learning from the team members. The pharmacists also completed multiple online training activities (Table 1). The MAC iOPS physician and a UofS faculty member, who is a board-certified psychiatric pharmacist, also provide ongoing mentorship to assist the pharmacists in developing expertise in this clinical area. The MAC iOPS team has the ability to directly refer patients to a psychiatrist (who is not a member of the MAC iOPS team) with specialization in addiction medicine who has agreed to take referrals from the program.

**Table 1:** Continuing education completed by MAC iOPS pharmacists

**Pain specific**
• American Society of Health-System Pharmacists (ASHP) Pain Management Certificate • College of Physicians and Surgeons of Saskatchewan Project ECHO Management of Chronic Pain sessions
**Opioid specific**
• University of British Columbia Continuing Professional Development Addiction Care and Treatment Online Course • University of Toronto Safer Opioid Prescribing Program • Saskatchewan Continuing Professional Development for Pharmacy Professionals Harm Reduction Primer and OAT Standards Course

## The MAC iOPS vs traditional interprofessional chronic pain clinics

Typically, interprofessional chronic pain clinics use a model in which a physician or NP with expertise in chronic pain initially assesses the patient.^9^ Other health professionals on the team may assist with collection of the patient history, provision of education and the development of care plans; however, the physician/NP leads the team and usually makes the final treatment decisions, especially for medications.

The primary innovation of MAC iOPS is that a pharmacist leads the team and completes the initial pain assessment and the collection of a detailed pain history. The MAC iOPS pharmacist assumes responsibility for discussing treatment options with the patient and for collaborating with the patient’s existing primary care provider to implement a patient-centred care plan for the management of the chronic pain. The MAC iOPS dietitian only sees selected patients referred by the MAC iOPS pharmacists. The MAC iOPS physician performs the role of a consultant and does not meet with most patients. The MAC iOPS pharmacists discuss the more complex cases with the MAC iOPS physician, and she provides perspective and guidance on diagnoses and treatment recommendations. For these cases, the pharmacist gathers the patient information and organizes it into a preliminary care plan so that the team physician can listen to a brief case presentation and quickly contribute to the assessment and treatment recommendations. The pharmacist may also collaborate and coordinate care with other health professionals who are not directly part of the MAC iOPS team but who may be part of the patient’s existing interprofessional team (e.g., community pharmacists, psychiatrists, physical therapists, dietitians, home care teams). This innovative, pharmacist-led, interprofessional approach allows for care to be provided in an integrated manner in community settings where there is limited access to specialized care.

## The patients at MAC iOPS

Individuals eligible for referral to the MAC iOPS include those living with chronic pain and/or who are taking prescribed opioids. The MAC iOPS is not intended to manage previously diagnosed opioid use disorder (OUD) in the absence of chronic pain (e.g., provision of opioid agonist therapy for OUD). However, as there is often overlap between opioid use for chronic pain and the development of OUD, referrals are assessed on a case-by-case basis such that individuals with concomitant poorly controlled chronic pain and OUD may be accepted. Any health professional can make a referral to the MAC iOPS, and patients can self-refer. It is essential that referred individuals have an ongoing relationship with a family physician or NP with whom the MAC iOPS team can collaborate, as the MAC iOPS does not take over prescribing of medications. Once the patient has achieved their pain- and function-related goals, they are discharged from the program and continue with their preexisting care team.

## MAC iOPS services provided

The MAC iOPS offers 2 types of services: 1) individual patient consultations and 2) case conferences with the patient’s family physician, NP and/or other health care team members (Table 2). Appointments may be virtual or in person. Referring primary care providers are asked to provide results of recent musculoskeletal and neurological exams along with recent random urine drug screen results. Patients are encouraged to complete an intake package prior to the first appointment, which includes demographic information, self-administered assessment tools (Table 3) and an overview of current and past pain management modality trials.

**Table 2:** MAC iOPS services

Individual patient consultation	Case conference
• MAC iOPS pharmacist obtains a detailed history from the patient and collects additional patient information from relevant sources, as needed. • MAC iOPS pharmacist provides the patient with education regarding chronic pain self-management and medications. • MAC iOPS physician is consulted for complex cases. • MAC iOPS pharmacist forwards a consult letter outlining pharmacological and nonpharmacological recommendations (if applicable) to the primary prescriber and referring provider. • Patient is followed by the MAC iOPS team to assist with implementation and/or monitoring of medication changes (e.g., opioid tapering). • MAC iOPS pharmacist provides ongoing patient education and support.	• Case information template outlining the key patient information desired by the MAC iOPS is sent to the referring provider to complete. • The completed case information template is forwarded by the referring provider to the MAC iOPS, who reviews the patient information prior to the case conference. • Referring provider meets with MAC iOPS pharmacist by telephone/videoconference for guidance on the care of an individual’s chronic pain management and/or opioid use. • MAC iOPS physician is available for case conferences as well. • Following the case conference, MAC iOPS pharmacist provides a written summary of the assessment and recommendations to the requesting provider.

MAC iOPS, Medication Assessment Centre Interprofessional Opioid Pain Service.

**Table 3:** Assessment tools/questionnaires

**Pain assessment tools**
Brief Pain Inventory (BPI)–short form Central Sensitization Inventory (CSI) Neuropathic Pain Questionnaire (DN4)
**Mental health/substance use disorder questionnaires**
Patient Health Questionnaire–9 (PHQ-9) General Anxiety Disorders–7 (GAD-7) Pain Catastrophizing Scale (PCS) Prescription Opioid Misuse Index (POMI) Clinical Global Impression (CGI) Scale

During the initial assessment, which typically occurs over several appointments, the pharmacist reviews the available information (from the referring provider and the intake package) and speaks with the individual to 1) obtain a detailed account of their pain experience; 2) gather a thorough history (medical conditions, medication regimen, family/social history); 3) review existing diagnostic tests/investigations/specialist consultations; 4) establish the individual’s goals, values and preferences as they relate to chronic pain management; and 5) build rapport. Through ongoing interactions, the pharmacist offers education about multimodal chronic pain management, the role of medications and strategies to avoid medication-related harms. Individuals at risk of opioid overdose are asked about their familiarity with take-home naloxone and informed about its risk mitigation benefit. When needed, arrangements are made to provide the individual with training and supplies for take-home naloxone.

Following the initial assessment, the pharmacist works with the patient to develop a care plan to optimize their pain management. This may include addition or changes to pain pharmacotherapy, development of an opioid tapering plan, or transition to opioid agonist therapy. The care plan also includes recommendations to promote mind and movement strategies as part of a multimodal approach. This may include referral to a physiotherapist or a counsellor. All documentation by the pharmacist is recorded in the patient’s electronic medical record (EMR), and treatment recommendations are communicated to the referring health professional and the patient’s primary provider (family physician and/or NP). The pharmacist collaborates with the patient, the referring provider and other involved health professionals regarding support and follow-up for the treatment plan prior to and throughout implementation.

As an alternative to individual patient consultations, family physicians, NPs and other health providers may schedule a case conference with the MAC iOPS team (including the physician). The case conferences are designed to provide the requesting clinician with support, education and treatment recommendations regarding chronic pain management in a particular case. Although patients do not always participate in case conferences, it is encouraged that they engage with the MAC iOPS pharmacist by way of an individual consultation before or after the case conference to support the provision of person-centred treatment recommendations.

## The future of the MAC iOPS

### Expansion of the interdisciplinary approach

In addition to medication management, interprofessional care for patients with chronic pain should include supporting self-management skills training, counselling and physical therapies.^10-12^ The MAC iOPS pharmacists encourage patients to engage in a multimodal approach and also assist in connecting patients with appropriate services to further incorporate mind and movement strategies. However, access to these services in the community is challenging and often cost-prohibitive for patients. Additional funding has been sought to integrate a physical therapist and social worker into the MAC iOPS to streamline access to these essential aspects of care.

### Additional MAC iOPS pharmacist training

The MAC iOPS team has quickly identified that the provision of clinical services for individuals living with chronic pain involves an immense amount of patient counselling and support. Thus, advanced training in motivational interviewing would be beneficial for the pharmacy team. Given the strong connection between pain and mental health, the need for training in trauma-informed care, mental health first aid and suicide risk assessment has been identified by the MAC iOPS pharmacists. The MAC iOPS pharmacists are able to access expert guidance on these topics through the MAC iOPS physician and psychiatric pharmacist. Furthermore, given the complexity of the work and a high risk for compassion fatigue, self-care training and additional support for the MAC iOPS team may also need to be explored.

### Program evaluation

During the first 11 months of operation, the MAC iOPS has received approximately 100 patient referrals. A detailed program evaluation is planned over 3 years to assess various aspects of the MAC iOPS. The evaluation includes surveys of the MAC iOPS patients to assess their experience of the care provided by the MAC iOPS team, as well as changes in various pain and quality-of-life parameters. Surveys are also sent to referring health care providers to assess their experience with the MAC iOPS and to obtain feedback for ongoing quality improvement purposes.

A detailed retrospective chart review will be conducted on all patients who have received care at the MAC iOPS. The chart review will capture data on demographic and clinical characteristics of the MAC iOPS patients, referral source/reason for referral and number and type of appointments (virtual vs in person). In addition, the chart review will capture data on pain and mental health assessments (Table 3), as well as medications, including total daily morphine equivalents, high-risk nonopioid medications (e.g., central nervous system depressants), adjunctive nonopioid analgesics and take-home naloxone kits. The chart review will also summarize information on the trajectory of patients throughout their experience with the MAC iOPS, including which team members participated in care and specialist referrals such as physical therapy and counselling. Furthermore, the research team plans to use existing linked administrative health databases within the Saskatchewan Ministry of Health to assess changes in objective clinical endpoints on the MAC iOPS patients, such as hospitalizations and emergency room visits.

Another component of the evaluation plan includes MAC iOPS team member journaling and focus groups that will provide qualitative information on the experiences of the MAC iOPS clinicians in this unique program.

If the model proves to be effective, it could be expanded and further tested within a community pharmacy or primary care setting.

## Conclusion

The MAC iOPS aspires to provide essential care for Saskatchewan residents living with chronic pain and/or at high risk for opioid-related harm. The innovative approach of the MAC iOPS aims to capitalize on the expertise of pharmacists working within an interprofessional team to increase access to chronic pain care and strengthen the capacity for chronic pain management within Saskatchewan. If the MAC iOPS model proves to be successful, it could be expanded to other jurisdictions across the country to help address the management of chronic pain and the opioid crisis. ■
